# Effect of alkaline hydrogen peroxide assisted with two modification methods on the physicochemical, structural and functional properties of bagasse insoluble dietary fiber

**DOI:** 10.3389/fnut.2022.1110706

**Published:** 2023-01-11

**Authors:** Mengying Luo, Cheng Wang, Chenshu Wang, Caifeng Xie, Fangxue Hang, Kai Li, Changrong Shi

**Affiliations:** ^1^College of Light Industry and Food Engineering, Guangxi University, Nanning, China; ^2^Provincial and Ministerial Collaborative Innovation Center for Sugar Industry, Nanning, China; ^3^Faculty of Science, Centre for Agriculture and the Bioeconomy, Queensland University of Technology, Brisbane, QLD, Australia

**Keywords:** sugarcane bagasse, insoluble dietary fiber, modification, physicochemical properties, structural properties, functional properties

## Abstract

Bagasse is one of major by-product of sugar mills, but its utilization is limited by the high concentration of lignin. In this study, the optimal alkaline hydrogen peroxide (AHP) treatment conditions were determined by the response surface optimization method. The results showed that the lignin removal rate was 62.23% and the solid recovery rate was 53.76% when bagasse was prepared under optimal conditions (1.2% H_2_O_2_, 0.9% NaOH, and 46°C for 12.3 h), while higher purity of bagasse insoluble dietary fiber (BIDF) was obtained. To further investigate the modification effect, AHP assisted with high-temperature-pressure cooking (A–H) and enzymatic hydrolysis (A–E) were used to modify bagasse, respectively. The results showed that the water holding capacity (WHC), oil holding capacity (OHC), bile salt adsorption capacity (BSAC), and nitrite ion adsorption capacity (NIAC) were significantly improved after A-H treatment. With the A–E treatment, cation exchange capacity (CEC) and BSAC were significantly increased, while WHC, OHC, and glucose adsorption capacity (GAC) were decreased. Especially, the highest WHC, OHC, BSAC and NIAC were gained by A–H treatment compared to the A–E treatment. These changes in the physicochemical and functional properties of bagasse fiber were in agreement with the microscopic surface wrinkles and pore structure, crystallinity and functional groups. In summary, the A–H modification can effectively improve the functional properties of bagasse fiber, which potentially can be applied further in the food industry.

## 1. Introduction

Sugarcane is an annual or perennial tropical and subtropical herbaceous plant. It is widely loved by consumers because of its sweetness and the presence of various nutrients that are very beneficial to human metabolism. Sugarcane is not only consumed in the fruit market but is also an important cash crop for the production of sugar worldwide (1). Sugarcane is mainly grown in Brazil, India, China, and so on, most of which is used for producing sugar, and the by-product is bagasse. It was found that about 700 million tons of bagasse were produced by sugar production factories worldwide every year (2). At present, bagasse is used for feed production or is discarded indiscriminately, which causes environmental pollution. To the best of our knowledge, bagasse can be used as a potential high-value ingredient in food products, such as dietary fiber.

Dietary fiber (DF) can neither be digested nor absorbed to produce energy. Therefore, it was once considered a “nutrient-free substance” and did not get enough attention for a long time. However, with the in-depth development of nutrition and related science, scientists found that DF has a very important physiological role, such as lower the risk of diabetes, obesity, and intestinal diseases (3). Thus, researchers started to add DF as a food ingredient to meet the growing demand for healthy products. According to water solubility, DFs can be divided into soluble dietary fibers (SDF) and insoluble dietary fibers (IDF). IDF is much more abundant than SDF in plant foods (4) and a good source of food bases with functional groups that can effectively bind to glucose, oil, water, and toxic metal ions (5). However, classic extraction and hydrolysis cannot fully expose these functional groups. Many physical, chemical, and biological methods have been applied to modify IDF from different biomass sources to obtain better functional and processing properties (6). According to Zhang et al., hydroxypropylation, carboxymethylation, and complex enzymes (cellulase and hemicellulase) have been reported to improve the water-holding capacity, α-amylase inhibition activity, glucose delay index, and bile acid salt binding capacity of coconut cake dietary fiber (7). Zhang et al. investigated the effect of modifications (Acetylated, cross-linking and cellulose-hydrolyzed) on the millet bran dietary fiber (8). Acetylated modified millet bran dietary fiber had the highest adsorption capacity of bile acid salt and cholesterol. The highest adsorption capacity of copper ions and nitrite ions was observed after cross-linking modification. The highest glucose delay index activity and α-amylase inhibition activity were observed for cellulose-hydrolyzed millet bran dietary fiber. Zhuang et al. reported that mixing alkaline hydrogen peroxide-treated sugarcane dietary fiber with pre-emulsified sesame oil resulted in a batter with improved texture and gave good organoleptic scores (9). It can be deduced that different modifications have different effects on the structural, physicochemical, and functional properties of the same dietary fiber. However, every modification method has its limitations. Therefore, it is of great interest to evaluate the effects of modification methods and select the appropriate method for a specific food need. Currently, there is no reported study on the effect of modification methods on bagasse insoluble dietary fiber.

As the bagasse of the sugar factory is dark gray, its fibrous hydration property is poor, and the high lignin content will affect the fermentation. Alkaline hydrogen peroxide (AHP) is reported to be a green and mild pretreatment that adds value to agricultural by-products and effectively removes lignin (10). However, no one has used this method to optimize the effect of bagasse delignification. Therefore, we first designed four variables (hydrogen peroxide concentration, sodium hydroxide concentration, temperature, and time) to conduct response surface optimization experiments to obtain the process conditions of low lignin content and high solid recovery.

High-temperature-pressure cooking (HTPC) treatment could remove the lignin of plant cell and partially degrades the hemicellulose and cellulose molecules (11). Enzymatic hydrolysis (EH) treatment could destroy one or more specific chemical chains of the cell wall depending on the type and source of the enzyme (12). All of these methods can alter the microstructure and composition of DFs, resulting in both desirable and undesirable effects on structural and functional properties (13). However, to our knowledge, there is no comparative information on the effect of AHP assisted with HTPC or EH on bagasse dietary fiber properties, respectively. Therefore, this study also deeply discussed AHP assisted with HTPC (A–H) and AHP assisted with EH (A–E) were used to modify bagasse, respectively, and then the changes in physicochemical, structural, and functional properties of bagasse dietary fiber before and after modification were evaluated.

## 2. Materials and methods

### 2.1. Materials

Bagasse was provided by a sugar production factory, East Sugar Company, located in Nanning province, China. The bagasse was washed in flowing tap water and dried in an oven for 48 h (DHG-90338S-III, Shanghai, China) at 60°C. Then samples were smashed and sieved through a 40 mesh sieve to obtain a bagasse powder and stored at room temperature until use. Hemicellulase (2 × 10^5^ U/g) was purchased from Macklin (Shanghai, China). Other chemicals used were of analytical grade.

### 2.2. Bagasse insoluble dietary fiber preparation by alkaline hydrogen peroxide treatment

BIDF was prepared with alkaline hydrogen peroxide following the previous study (14) with modifications. Concentrations of 0.6, 0.9, and 1.2% (w/v, NaOH) and 0.5, 1.0, and 1.5% (w/v, H_2_O_2_) were prepared for the pretreatment of 1 g of bagasse with a solid loading of 1:60. This means that 1 g of bagasse was added to 60 mL of the solution containing the alkaline peroxide. The mixture was agitated continuously using a magnetic stirring water bath at 120 rpm, and the temperature and the reaction time were set to the desired degree of 35°C, 45°C, or 55°C for 8, 12, or 16 h. The material was collected by filter and washed with distilled water until neutralized, freeze-dried for 36 h, and finally weighed to obtain the solids recovery. Finally, it was milled and passed the sieved (60 mesh) to obtain powders, and BIDF was obtained. Lignin content was obtained by a two-step acid hydrolysis method. The combination of acid-soluble and acid-insoluble lignin constitutes the total lignin concentration. The percent lignin removal and percent solid yield was calculated as follows:


(1) $$\begin{array}{c} percent\;lignin\;removal\;\left(\%\right)\;=\;\frac{W_{0}-W_{1}}{W_{0}}\times 100 \end{array}$$

(2) $$\begin{array}{c} percent\;solid\;yield\;\left(\%\right)\;=\;\frac{M_{1}}{M_{0}}\times 100 \end{array}$$

where W_0_ and W_1_ are the percentages of total lignin content before and after AHP treatment, respectively; M_0_ and M_1_ are the weights of dry samples before and after AHP treatment, respectively.

### 2.3. AHP assisted with two modification methods

#### 2.3.1. AHP assisted with high-temperature-pressure cooking (A–H)

2 g BIDF and 60 mL distilled water (1:30, w/v) were added to the pressure-resistant tube, well mixed, and placed in a capacitive pressure steam sterilizer (XYR20, Zhejiang, China). The temperature was set to 120°C for 1 h. After the high-temperature-pressure cooking treatment, the material in the pressure-resistant tube was poured into Petri dishes and subsequently freeze-dried for 36 h. Finally, the freeze-dried samples were milled and passed through a 60-mesh sieve. H-BIDF was obtained.

#### 2.3.2. AHP assisted with enzymatic hydrolysis (A–E)

10 g BIDF was mixed with 400 mL distilled water (1:40, w/v) in a conical flask and adjusted to pH = 5.5 with 0.1 M acetic acid solution. Then, 0.5 g hemicellulase was added and the mixture was placed in a shaker and reacted at 50°C for 1 h. The hydrolysis reaction was stopped by heating at 95°C for 10 min and washed with distilled water until neutralized. After that, it was freeze-dried, milled, and sieved, and E-BIDF was obtained.

### 2.4. Approximate chemical composition and physical properties

The chemical composition of the bagasse and its derivatives (BIDF, H-BIDF and E-BIDF) were determined by AOAC official methods (15), including moisture (method 925.09), total dietary fiber (TDF), SDF, IDF (method 991.43) and ash (method 942.05). The contents of cellulose, hemicellulose, and lignin were determined as described by Liu et al. (16). Briefly, the samples were first subjected to two-step acid hydrolysis, and sand core filtration and the residue obtained was dried to constant weight, cauterized to obtain the acid-insoluble lignin content, and the filtrate was measured at UV absorption 240 nm to obtain the acid-soluble lignin concentration. Subsequently, the concentration of sugars in the hydrolysate was determined by high-performance liquid chromatography (LC 1260 ll, Agilent, USA, equipped with a Shodex SP0810 column and guard column) and converted to obtain the concentrations of cellulose and hemicellulose. The column temperature was 80°C, the mobile phase was ultra-pure water, the flow rate was 0.5 mL/min, and the run time was 50 min.

The color was measured using a spectrophotometer (CS-420, CHNSpec technology, China) to obtain the values of L, a, and b. “L” represents the brightness of the object: 0-100 means from black to white, “a” represents the red-green color of the object: a positive value indicates red, a negative value indicates green, “b” represents the yellow-blue color of the object: positive values indicate yellow, negative values indicate blue. The whiteness index (WI) was calculated as the following equation:


(3) $$\begin{array}{c} WI\;=\;100-\sqrt{{\left(100-L\right)}^{2}+a^{2}+b^{2}} \end{array}$$

### 2.5. Structural properties

#### 2.5.1. Scanning electron microscopy

The samples dried to constant weight were directly glued to the conductive adhesive and sprayed with gold for 45 s using an Oxford Quorum SC7620 sputter coater at 10 mA; the sample morphology was subsequently photographed using a ZEISS Sigma 300 scanning electron microscope at a voltage of 3 kV.

#### 2.5.2. Fourier-transform infrared spectroscopy

The changes in molecular structure were analyzed using an infrared spectrometer (Nicolet 670, USA). Four samples dried to constant weight were mixed completely with potassium bromide (1:100, w/w), then placed in a mold and pressed into transparent sheets on a hydraulic press, and finally, the samples were tested in an infrared spectrometer. The FT-IR spectral wave number was in the range of 400–4,000 cm^−1^ with a scan number of 32 and a resolution of 4 cm^−1^.

#### 2.5.3. X-ray diffraction

The XRD patterns of four samples were obtained by a Bruker D8 Advanced diffractometer operating at 40 kV and 30 mA. The diffraction angle (2θ) was in the 10°-60° range using a step size of 0.02° and the scanning rate was 1.2°/min. The crystallinity index (CI) was calculated by the classic formula:


(4) $$\begin{array}{c} CI\left(\%\right)\;=\;\frac{I_{002}-I_{AM}}{I_{002}}\times 100 \end{array}$$

where I_AM_ is the lowest intensity of the peak at the angle of around 18° and I_002_ was the maximum intensity of the peak located at the angle of 22°.

### 2.6. Physicochemical and functional properties

#### 2.6.1. Water holding capacity

WHC was determined by the method (17) with some modifications. An appropriate amount of dietary fiber was first weighed, saturated with excess water, shaken, and allowed to stand at room temperature for 2 h. The excess water was then filtered off using a 700 mesh filter cloth, a portion of which was carefully removed from the wet sample, weighed, and dried to a constant weight in a hot air drying oven (105°C). The WHC was calculated as the following equation:


(5) $$WHC\;\left(g/g\right)\;=\;\frac{M_{1}-M_{2}}{M_{2}}$$

where M_1_ and M_2_ are the weights of the wet and dry samples, respectively.

#### 2.6.2. Oil holding capacity

OHC was determined by the method (18) with some modifications. Firstly, 0.25 g DF samples and 10 mL corn oil were added into 50 mL centrifuge tubes. Then, they were mixed well and left at room temperature for 4 h, centrifuged at 1,144 g for 20 min. Finally, the excess oil was discarded and the residues were weighed. The OHC was calculated as the following equation:


(6) $$\begin{array}{c} OHC\;\left(g/g\right)\;=\;\frac{M_{1}-M_{2}}{M_{2}} \end{array}$$

where M_1_ and M_2_ are the weights of the wet and dry samples, respectively.

#### 2.6.3. Cation exchange capacity

The CEC was based on the methods (19) with some modifications. 1 g IDF sample was weighed in a conical flask, mixed with 0.1 M hydrochloric acid at a stock-liquid ratio of 1:70 (w/v), and acidified with magnetic stirring at room temperature for 24 h. The fibers were then washed with distilled water until the wash was free of Cl^−^ (detected by 10% AgNO_3_ solution), and the solid residue was freeze-dried for 36 h. Disperse 0.1 g of acidified dried sample in 10 mL of 5% NaCl solution and stir magnetically at room temperature for 2 h. Add 1 drop of phenolphthalein indicator and titrate with 0.01 M NaOH until the color of the solution turns slightly red and read the volume of NaOH solution consumed. The cation exchange capacity was expressed as the amount of NaOH consumed per g of sample. The CEC was calculated as the following equation:


(7) $$\begin{array}{c} \text{CEC}\;\left(\text{mmol}/\text{g}\right)\;=\;\frac{0.01({\text{V}}_{1}-{\text{V}}_{2})}{\text{M}} \end{array}$$

where V_1_ is the volume of sodium hydroxide consumed by titrating the dietary fiber sample (mL); V_2_ is the volume of sodium hydroxide consumed by titrating the blank (mL); M is the mass of the dietary fiber sample.

#### 2.6.4. Glucose adsorption capacity

The GAC was based on the method (20) with some modifications. Briefly, 0.5 g of IDF sample and 50 mL of glucose solution (50 mmol/L) were mixed well in a conical flask, which was placed under adsorption in a shaker at 37°C for 6 h and centrifuged at 3,000 g for 10 min. 1 mL of supernatant was transferred into a test tube and then mixed with 2.0 mL of dinitro-salicylate (DNS) reagent. The mixture was incubated in boiling water for 5 min and cooled flowing water to room temperature. Finally, add 9 mL of distilled water into the tube and mix well. The residual content of glucose was measured by the absorbance value of the solution at 540 nm by InfiniteM200PRO (Tecan, Austria) and quantified based on the standard curve. The GAC was calculated as the following equation:


(8) $$\begin{array}{c} GAC\;\left(mg/g\right)\;=\;\frac{M_{1}-M_{2}}{M} \end{array}$$

where M_1_ is the mass of glucose in the solution before adsorption, M_2_ is the mass of glucose in the solution after adsorption, and M is the mass of the dietary fiber sample.

#### 2.6.5. Bile salt adsorption capacity

The BSAC was based on the method (21) with some modifications. 0.1 g of IDF sample was added to the conical flask, and then 5 mL of sodium cholate phosphate buffer (pH = 7) was added to mimic the small intestinal environment. Adsorbed in a shaker at 37°C for 2 h, centrifuged at 4,000 g for 10 min. 1 mL of supernatant was taken in a corked test tube, and 6 mL of 45% sulfuric acid and 1 mL of 0.3% furfural were added sequentially. Mix well and place the reaction in a constant temperature water bath at 65°C for 30 min. Immediately after the reaction, the reaction was cooled in an ice water bath, and the absorbance value was measured at 620 nm using InfiniteM200PRO (Tecan, Austria) and compared with the standard curve. The formula was calculated as follows:


(9) $$\begin{array}{c} BSAC\;\left(mg/g\right)\;=\;\frac{M_{1}-M_{2}}{M} \end{array}$$

Where M_1_ is the mass of sodium cholate in the solution before adsorption, M_2_ is the mass of sodium cholate in the solution after adsorption, and M is the mass of the dietary fiber sample.

#### 2.6.6. Nitrite ion adsorption capacity

The NIAC was based on the method (22) with some modifications. 0.1 g of IDF sample was mixed with 10 mL of NaNO_2_ solution (10 μg/mL) with the pH adjusted to 2.0 and 7.0, simulating the stomach and small intestinal environment. The mixture was incubated at 37°C for 2 h, then centrifuged at 1,144 g for 10 min. The supernatant (1 mL) was transferred into a colorimetric tube, mixing with 2 mL p-aminobenzene sulfonic acid (0.4%, w/v) and 1 mL hydrochloride naphthodiamide (0.2%), and then ultra-pure water was added to the tube until the volume reached to 50 mL. After the mixture was reacted in the dark for 15 min, the residual content of glucose was measured by the absorbance value of the solution at 538 nm by InfiniteM200PRO (Tecan, Austria) and quantified based on the standard curve. The NIAC was calculated as the following equation:


(10) $$\begin{array}{c} NIAC\;\left(\text{μ}g/g\right)\;=\;\frac{M_{1}-M_{2}}{M} \end{array}$$

where M_1_ is the mass of NaNO_2_ in the solution before adsorption, M_2_ is the mass of NaNO_2_ in the solution after adsorption, and M is the mass of the dietary fiber sample.

### 2.7. Statistical analyses

All experiments were performed in triplicate and the results were expressed as mean ± standard deviation (SD). Statistical analysis was carried out by IBM SPSS statistical software (version 27.0, SPSS Inc., Chicago, IL, USA). *P* < 0.05 was considered to be statistically significant.

## 3. Results and discussion

### 3.1. Response surface optimization experimental results

It was reported that strong oxidants produced by hydrogen peroxide under suitable alkaline conditions cause lignin oxidation and biomass depolymerization (23). Cabrera et al. concluded that there was no significant change in biomass structure under low alkaline conditions (24). In contrast, high alkaline conditions lead to a significant dissolution of hemicellulose, reducing the recovery of cellulose from insoluble material. Thus, the use of response surface design for AHP process optimization of bagasse is essential.

In this experiment, we used 4 factors and 3 levels to evaluate the effect of AHP delignification, and the results of each experiment are shown in Table 1. The percent lignin removal ranged from 48.12 to 68.71 and the percent solid yield ranged from 40.6 to 64.7. The response equations for lignin removal and solids recovery were obtained based on the analysis of the data, where X_1_, X_2_, X_3_, and X_4_ are time, temperature, sodium hydroxide concentration and peroxide concentration, respectively.

**Table 1:** The design and results of the Box-Behnken experiment.

**Run**	**X_1_: Time(h)**	**X_2_:** **Temperatuere** **(°C)**	**X_3_: NaOH(w/v)**	**X_4_: H_2_O_2_(w/v)**	**Response 1: Percent lignin removal (%)**	**Response 2: Percent solid yield (%)**
1	16	55	0.9	1	67.93	41.5
2	16	45	0.6	1	62.7	46.3
3	12	45	1.2	1.5	61.2	47.6
4	12	45	1.2	0.5	61.89	48.7
5	8	45	0.9	1.5	50.76	60.8
6	12	55	1.2	1	68.71	40.6
7	16	45	0.9	1.5	60.86	52.32
8	8	45	0.9	0.5	56.42	56.4
9	12	45	0.9	1	63.5	54.5
10	16	35	0.9	1	61.48	49.7
11	12	45	0.9	1	62.03	54.15
12	12	55	0.9	1.5	60.59	52.4
13	12	55	0.9	0.5	62.47	51.1
14	8	55	0.9	1	57.77	50.5
15	12	45	0.9	1	65	47.3
16	12	55	0.6	1	61.4	52.1
17	12	35	0.6	1	48.5	57.4
18	8	35	0.9	1	51.98	58.2
19	12	45	0.6	1.5	49.4	64
20	8	45	0.6	1	52.21	58.21
21	12	35	0.9	1.5	48.12	64.7
22	12	35	1.2	1	59.81	49.9
23	16	45	0.9	0.5	62.5	46.7
24	12	45	0.9	1	62.4	53.7
25	12	45	0.9	1	62.07	54.14
26	12	45	0.6	0.5	57.5	52.7
27	16	45	1.2	1	67.63	41.2
28	8	45	1.2	1	62.92	45.3
29	12	35	0.9	0.5	55.4	55.1

Percent lignin removal (%) = −52.94698 + 2.75083X_1_ + 2.43963X_2_ + 51.56389X_3_ + 1.43667X_4_ +0.004125X_1_X_2_ – 1.20417X_1_X_3_ + 0.5025X_1_X_4_ – 0.3333X_2_ X_3_ + 0.27X_2_X_4_ + 12.35X_3_X_4_ – 0.05382812X${}_{1}^{2}$ – 0.022363X${}_{2}^{2}$ – 11.36111X${}_{3}^{2}$ – 17.47X${}_{4}^{2}$

Percent solid yield (%) = 22.13619 + 0.892875X_1_ + 0.622225X_2_ + 53.94111X_3_ + 13.62033X_4_ – 0.003125X_1_X_2_ + 1.62708X_1_ X_3_ + 0.1525X_1_X_4_ – 0.333333X_2_ X_3_ – 0.415X_2_X_4_ – 20.66667X_3_X_4_ – 0.143583X${}_{1}^{2}$ – 0.002886X_2_^2^ – 29.85926X${}_{3}^{2}$ + 13.50567X${}_{4}^{2}$

**Figure 1:**
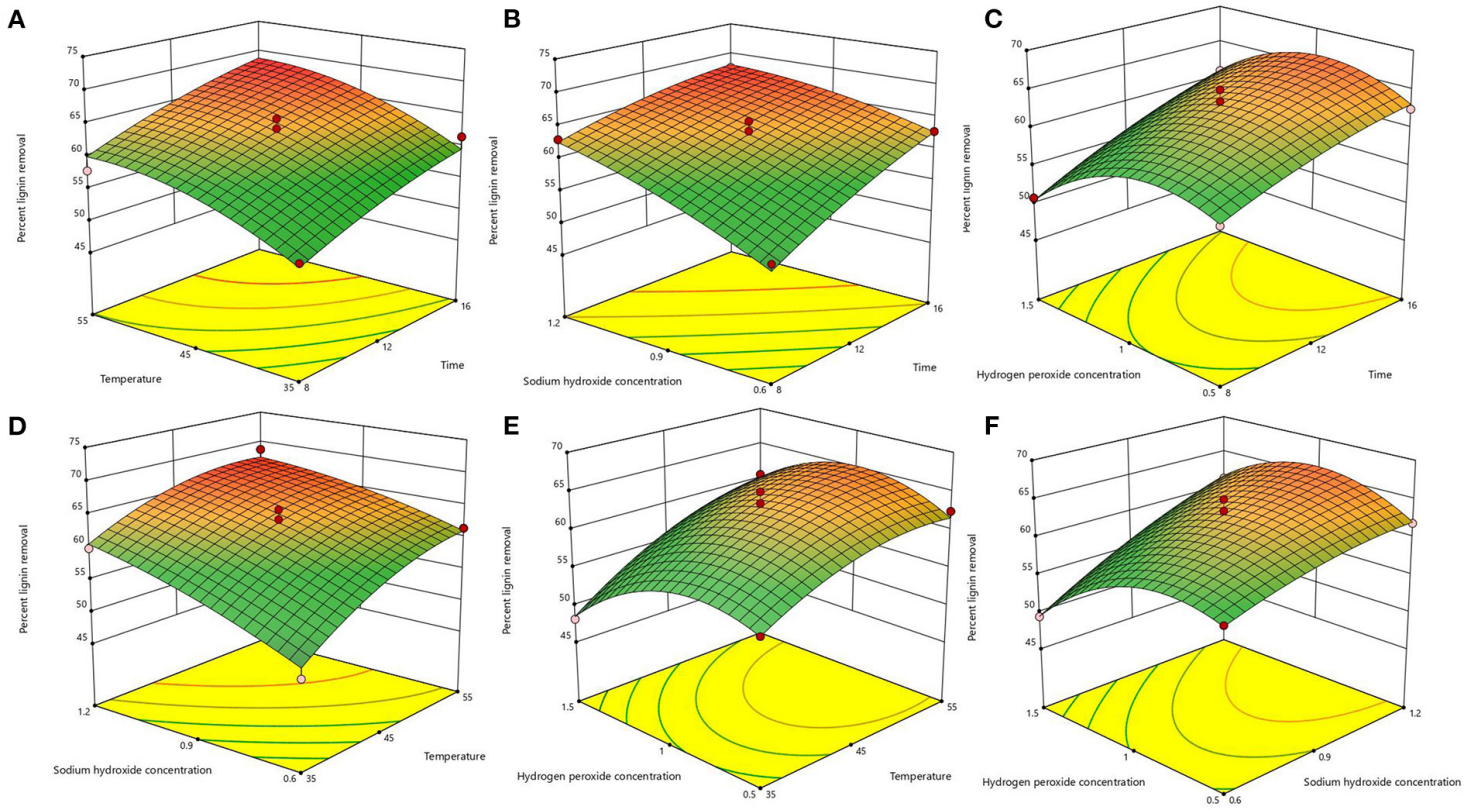
Response surface plots of the interaction for lignin removal. **(A)** temperature and time; **(B)** sodium hydroxide concentration and time; **(C)** hydrogen peroxide concentration and time; **(D)** sodium hydroxide concentration and temperature; **(E)** hydrogen peroxide concentration and temperature; **(F)** hydrogen peroxide concentration and sodium hydroxide concentration.

**Figure 2:**
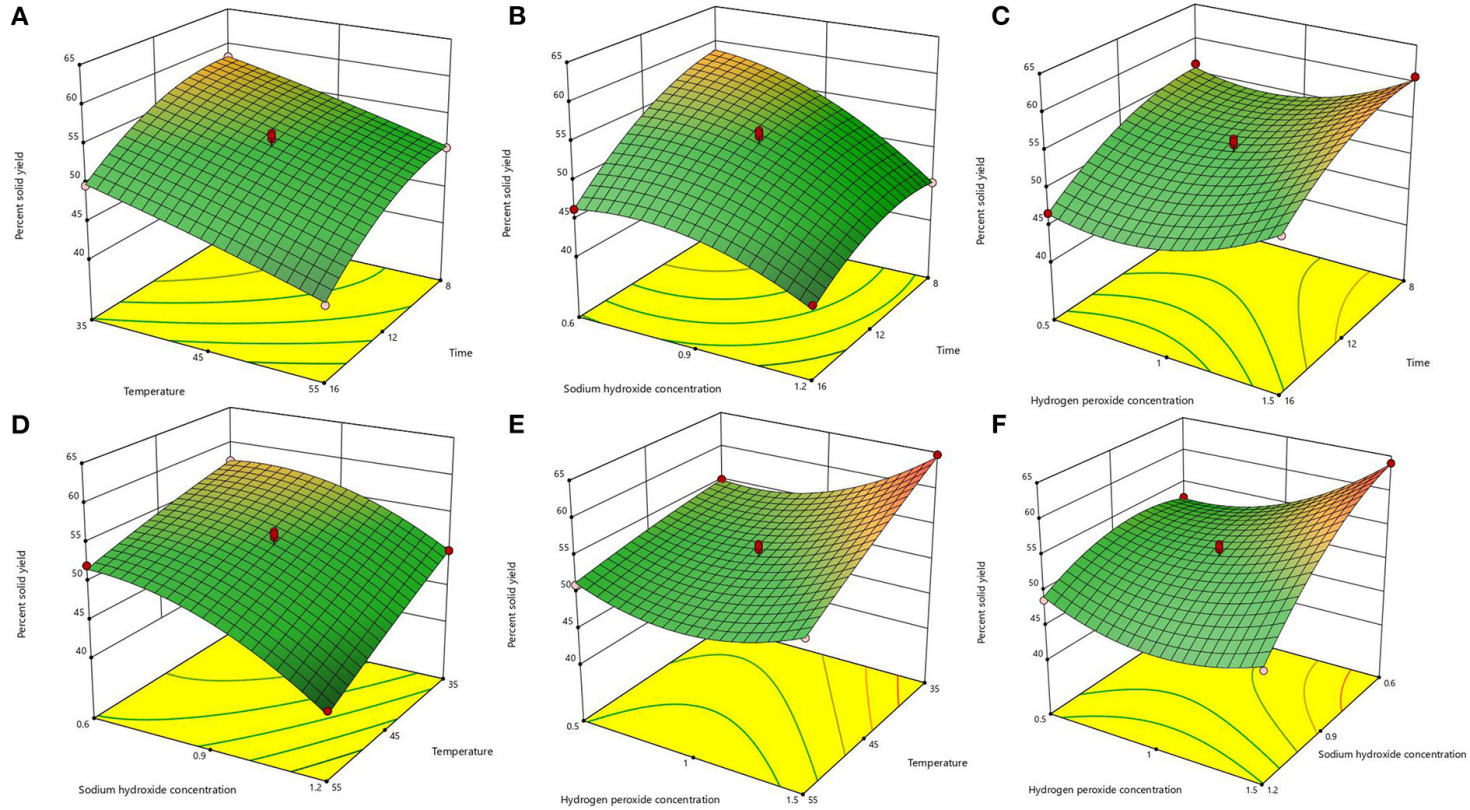
Response surface plots of the interaction for solid yield. **(A)** temperature and time; **(B)** sodium hydroxide concentration and time; **(C)** hydrogen peroxide concentration and time; **(D)** sodium hydroxide concentration and temperature; **(E)** hydrogen peroxide concentration and temperature; **(F)** hydrogen peroxide concentration and sodium hydroxide concentration.

After the analysis by Design-Expert 13.0 software, it is known that this equation is a suitable mathematical model for each parameter of the AHP treatment process, so this regression equation can be used to determine the best treatment process for alkaline hydrogen peroxide. The response surface plots of lignin removal rate and solids yield are shown in Figures 1, 2. If the curve is steeper, it indicates that the factor has more influence on the response value. The degree of influence of the four experimental factors on the lignin removal rate was temperature > time > sodium hydroxide concentration > hydrogen peroxide concentration. And the degree of influence on solid recovery was sodium hydroxide concentration > time > temperature > hydrogen peroxide concentration.

The optimal process conditions for bagasse pretreatment were obtained as a treatment time of 12.26 h, a temperature of 46.003°C, a sodium hydroxide concentration of 0.85%, and a hydrogen peroxide concentration of 1.19%. Considering the operability, the optimal conditions were adjusted to 12.3 h treatment time, 46°C temperature, 0.9% sodium hydroxide concentration, and 1.2% hydrogen peroxide concentration, and three validation tests were conducted under these optimal conditions, and the lignin removal rate of bagasse was 62.23% and the solid yield was 53.76%, which were similar to the predicted values of the model. This indicates that the model predicts accurate parameters and has a certain guiding significance for practical operation.

### 3.2. Effect on approximate chemical composition and color

The proximate composition and color of the four samples were listed in Table 2. Bagasse treated with optimized AHP reduced acid-insoluble lignin and acid-soluble lignin content by 71.85 and 20.04%, respectively, and significantly increased IDF, SDF, TDF, and cellulose content (*P* < 0.05). In the food industry, color is one of the important parameters of food ingredients and affects the acceptability of food products by consumers (25). In addition, changes in food color help us to distinguish changes in the physical and chemical properties of raw food materials (26). The WI of bagasse increased from 67.69 to 84.05, which can significantly increase consumer acceptance. Compared to BIDF, although H-BIDF and E-BIDF showed a decrease in whiteness, the ash removal rate was significantly higher, 96.15 and 93.75%, respectively. Besides, the cellulose, hemicellulose, and content of H-BIDF were significantly decreased (*P* < 0.05). However, the cellulose content of E-BIDF increased and the hemicellulose content decreased by 80.17% and 2.06%, respectively. This indicates that HTPC treatment degrades some cellulose and hemicellulose of BIDF, while hemicellulase significantly degrades hemicellulose of BIDF, which may help dietary fiber to expose more functional groups and better perform its functional properties *in vivo*.

**Table 2:** The approximate chemical composition and color of bagasse insoluble dietary fiber modified by different methods.

**Sample**	**Bagasse**	**BIDF**	**H-BIDF**	**E-BIDF**
Moisture (g/100 g)	7.26 ± 0.04^a^	7.14 ± 0.33^a^	7.21 ± 0.02^a^	6.64 ± 0.08^b^
IDF (g/100 g)	86.77 ± 0.71^c^	95.52 ± 0.23^a^	95.46 ± 0.13^a^	91.76 ± 0.13^b^
SDF (g/100 g)	0.27 ± 0.06^d^	0.53 ± 0.02^c^	0.86 ± 0.07^a^	0.70 ± 0.01^b^
TDF (g/100 g)	87.04 ± 0.65^c^	96.04 ± 0.20^a^	96.32 ± 0.07^a^	92.46 ± 0.14^b^
Cellulose (g/100 g)	38.20 ± 0.71^d^	68.90 ± 0.99^b^	63.65 ± 0.35^c^	72.65 ± 0.49^a^
Hemicellulose (g/100g)	18.55 ± 0.49^a^	17.65 ± 0.64^a^	15.25 ± 0.64^b^	3.50 ± 0.00^c^
Acid-insoluble lignin (g/100 g)	22.98 ± 1.05^a^	6.47 ± 0.33^c^	7.09 ± 0.37^c^	10.28 ± 0.45^b^
Acid-soluble lignin (g/100 g)	5.24 ± 0.04^a^	4.19 ± 0.15^b^	4.00 ± 0.01^b^	3.20 ± 0.04^c^
Ash (g/100 g)	2.07 ± 0.13^a^	2.08 ± 0.10^a^	0.08 ± 0.00^b^	0.13 ± 0.01^b^
L	70.43 ± 0.13^d^	88.24 ± 0.07^a^	83.57 ± 0.04^c^	85.35 ± 0.19^b^
a	2.73 ± 0.01^a^	−0.06 ± 0.15^d^	0.59 ± 0.17^b^	0.22 ± 0.03^c^
b	12.74 ± 0.04^a^	10.77 ± 0.13^c^	12.14 ± 0.07^b^	9.89 ± 0.10^d^
WI	67.69 ± 0.11^d^	84.05 ± 0.14^a^	79.56 ± 0.08^c^	82.32 ± 0.11^b^

Different letters (a, b, c, d) denote significantly different in the column (*p* < 0.05).

### 3.3. Structural properties

#### 3.3.1. Scanning electron microscopy

**Figure 3:**
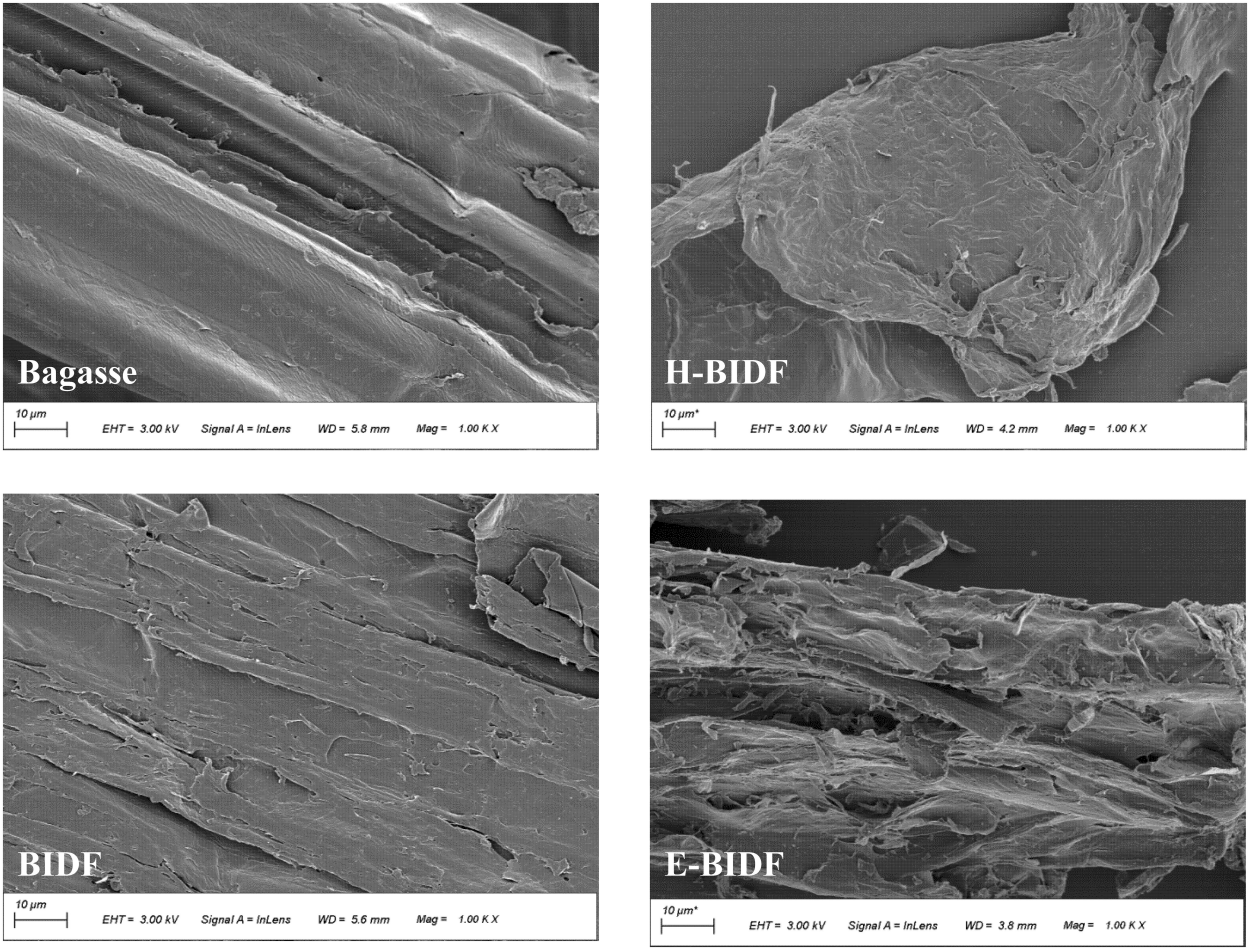
The SEM images of bagasse, BIDF, H-BIDF and E-BIDF at ×1,000 magnification.

The SEM images of the bagasse and its modified derivatives (BIDF, H-BIDF and E-BIDF) are shown in Figure 3. Each sample had different morphological characteristics. It was found that bagasse were long strips of fibers and the structure was relatively flat and complete, which is consistent with the previous study (27). After AHP treatment, striated cracks were formed on the BIDF surface, which could be due to lignin degradation. Compared with BIDF, the surface of H-BIDF became rougher and formed water ripple-like folds and pore layers, which might have a positive impact on the functional properties of the sample. The structural integrity of E-BIDF was destroyed and its surface was uneven, forming a large number of honeycomb microstructures, which may be due to the massive degradation of wall polysaccharides caused by hemicellulase. Numerous studies have shown that the irregular surface and porous structure contribute to the adsorption capacity of the material (28), which is crucial for the subsequent adsorption of ions and molecules. Nevertheless, this special structure could increase the relative surface area, and lead to the increase of WHC, OHC, BSAC, NIAC.

#### 3.3.2. Fourier-transform infrared spectroscopy

**Figure 4:**
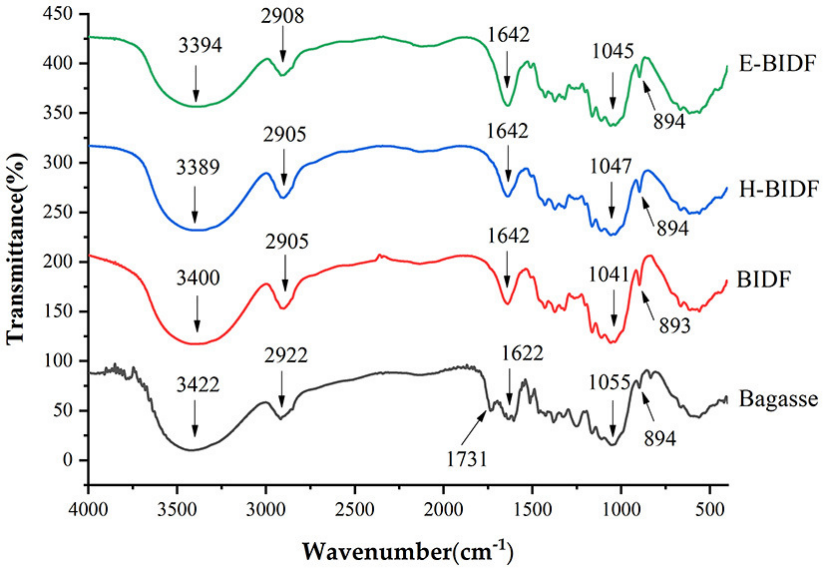
The FT-IR images of bagasse, BIDF, H-BIDF and E-BIDF.

The FT-IR images of the bagasse with its modified derivatives (BIDF, H-BIDF, and E-BIDF) were recorded from 400 to 4,000 cm^−1^ in Figure 4. It can be seen from the figure that the four fiber samples have similar characteristic peaks and show common fingerprints of lignocellulose fibers (29). The wide band positioned around 3,422 cm^−1^ was mainly due to the extension of O-H bound to hydrogen and hydroxyl groups produced by cellulose and hemicellulose (30). Compared with bagasse, the three modified bagasse dietary fibers had obvious blue shifts at the frequency band of 3,422 cm ^−1^, which indicated that their organic molecules of hydrogen bonding may be partly destroyed (31). The wide band was positioned around 2,922 cm^−1^ to represent the aliphatic saturated C-H stretching vibration in cellulose and hemicelluloses or the stretching vibration peak of -OH in the hemicellulose molecule or intermolecular. The characteristic peak of C=O near 1,731 cm^−1^ indicates that the AHP pretreatment of bagasse effectively degraded the C=O of the fiber. The peak around 1,622 cm^−1^ is the characteristic peak of the lignin benzene ring, which indicates a redshift after the three treatments. In addition, it can be seen that the peak intensity of E-BIDF increases significantly, which is attributed to the large reduction of hemicellulose and the relative increase of lignin content after enzymatic hydrolysis. The peak at 1,055 cm^−1^ was assigned to the C-OH stretching and β-glycosidic linkages of the cellulose glucose ring. The absorption peak with an intensity near 890 cm^−1^ represents the breakage of β-glycosidic bonds, which can be seen to be the most pronounced after HTPC treatment. According to FT-IR spectra, bagasse fibers were modified in three ways and their chemical structures were disrupted to different degrees.

#### 3.3.3. X-ray diffraction

**Figure 5:**
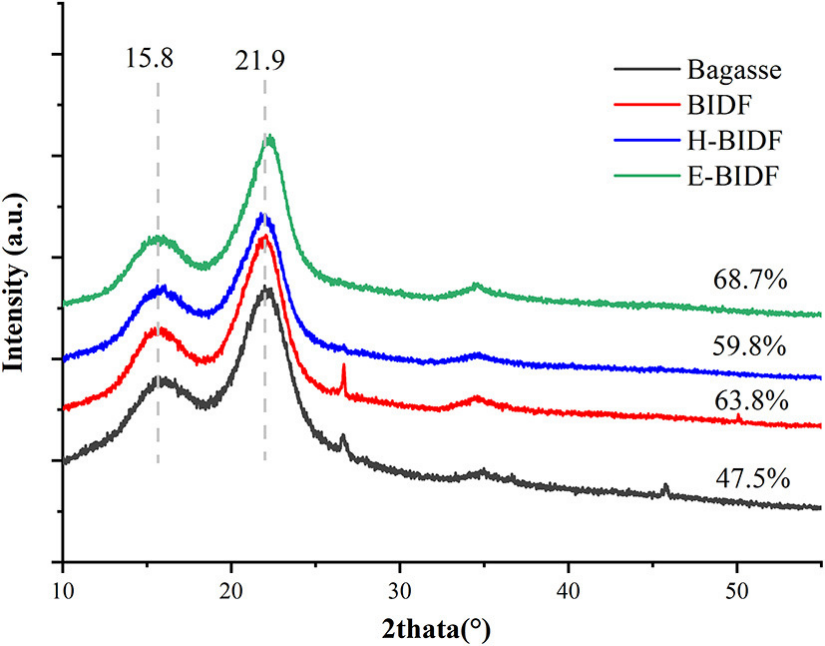
The XRD patterns of bagasse, BIDF, H-BIDF and E-BIDF.

The XRD patterns of the bagasse and its derivatives (BIDF, H-BIDF and E-BIDF) were illustrated in Figure 5. Cellulose has a crystalline structure, in contrast to lignin and hemicellulose (amorphous) (32). As can be seen in the figure, all the samples had the characteristic crystalline peaks at 15.8° and 21.9° 2θ, indicating that they are all double helix type I cellulose (33). The CI of bagasse was 47.5%, and after AHP treatment, the CI of BIDF was significantly increased to 63.8%, similar to the results of the ginseng dietary fiber study (34). After HTPC treatment of BIDF, the CI of H-BIDF (59.8%) was significantly decreased, which indicated that a portion of the cellulose crystalline region was destroyed. It may be the effect of HTPC to promote fiber dissolution (35), as in Table 2 a significant increase in soluble dietary fiber content. However, after being treated by the enzymatic digestion of hemicellulose, the CI of E-BIDF (68.7%) was increased, which was due to the degradation of hemicellulose. The result suggested that enzymatic treatment of bagasse dietary fiber was helpful to improve the structural stability of cellulose.

### 3.4. Physicochemical and functional properties

#### 3.4.1. Water holding capacity and oil holding capacity

**Figure 6:**
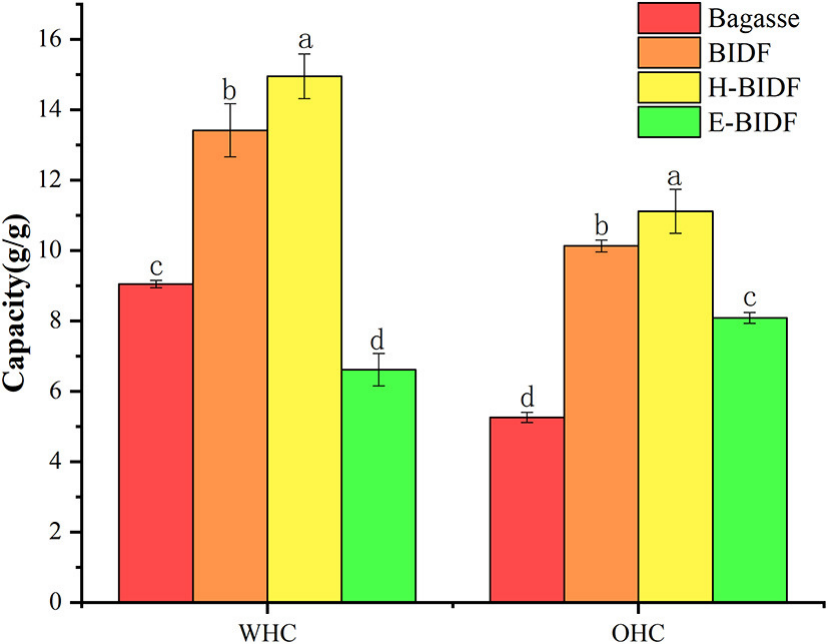
The WHC and OHC of bagasse, BIDF, H-BIDF and E-BIDF. Different letters (a, b, c, d) denote significantly different in the column (*p* < 0.05).

The WHC and OHC of the bagasse and modified derivatives were shown in Figure 6. It was reported that insoluble fiber with high WHC can accelerate the speed of defecation, reduce the pressure in the rectum and urinary system, relieve the symptoms of urinary system diseases and prevent constipation (36), and enable the rapid discharge of toxic substances from the body. IDF can adsorb oil and other components through hydrophobic interaction and capillary adsorption, helping the body remove excess fat, and thereby reducing the risk of chronic diseases (37). This study found that the amount of lignin was negatively correlated with WHC and OHC, while the amount of hemicellulose would be positively correlated. The results for WHC were similar to those of Qi et al. (18), but the opposite conclusion for OHC may be due to the presence of non-dietary fiber impurities in rice bran dietary fiber. H-BIDF showed the highest WHC (14.95 g/g) and OHC (11.12 g/g), mainly due to the significant degradation of lignin by the AHP process, in addition to the possibility that the combined HTPC treatment disrupted the intermolecular forces of DF.

#### 3.4.2. Cation exchange capacity

**Figure 7:**
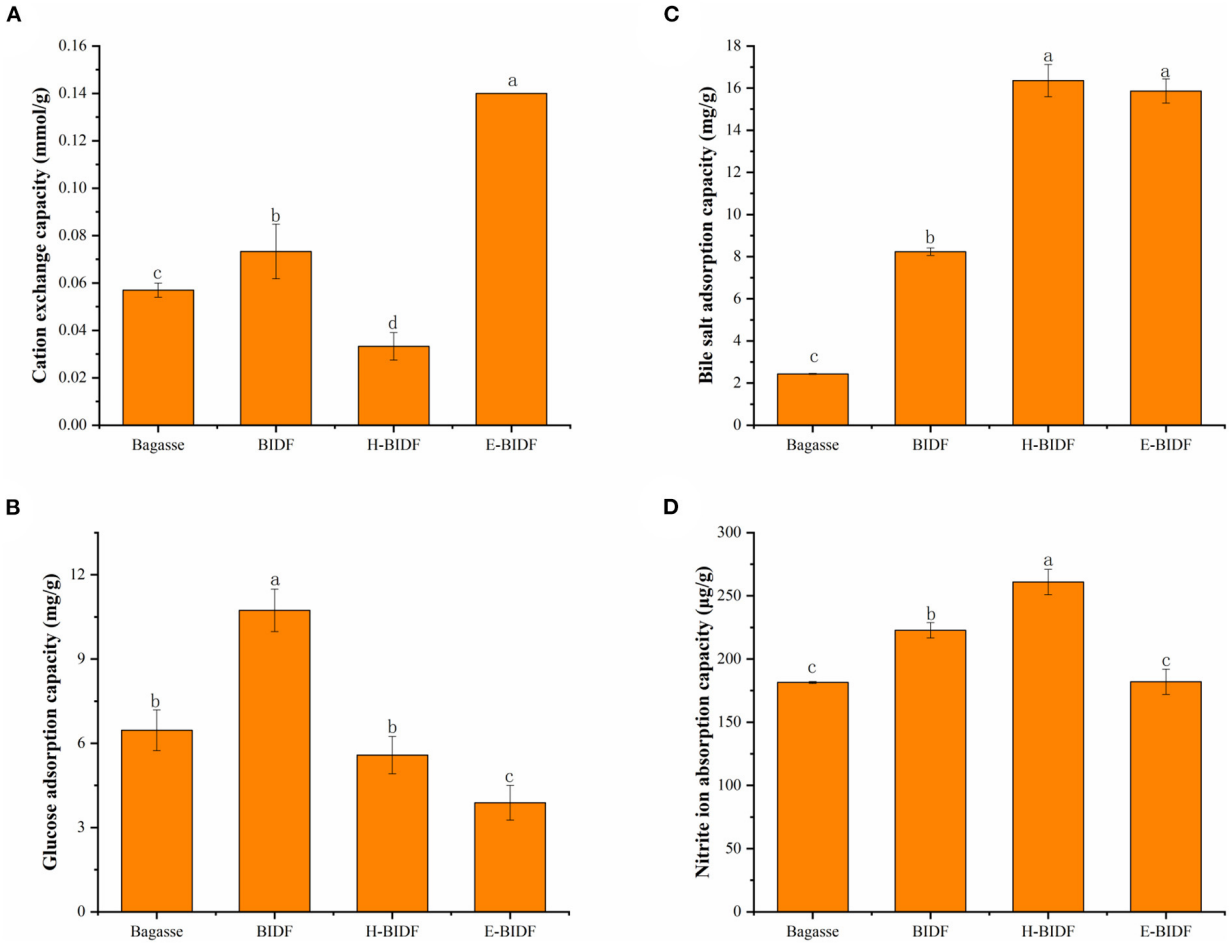
The CEC **(A)**, GAC **(B)**, BSAC **(C)**, and NIAC **(D)** of bagasse, BIDF, H-BIDF and E-BIDF. Different letters (a, b, c, d) denote significantly different in the column (*p* < 0.05).

The chemical structure of dietary fiber contains some carboxyl and hydroxyl side chain groups that can be reversibly exchanged with organic cations, thus affecting the pH value of the digestive tract and creating a more retentive environment for digestion and absorption (38). Results in Figure 7A suggested that bagasse treated with optimized alkaline hydrogen peroxide significantly increased the CEC by 28.6%. In addition, the BIDF treated with hemicellulase hydrolysis significantly improved the CEC of BIDF (*P* < 0.05). It was reported that the CEC was mainly related to the anion-bearing functional groups such as carboxyl and hydroxyl phenolic groups on the fiber (39), which may be the main reason for the increased CEC of E-BIDF (0.14 mmol/g). On the contrary, the hydrothermal reaction of HTPC may destroy its carboxyl and hydroxyphenyl groups, which harmed its CEC.

#### 3.4.3. Glucose adsorption capacity

GAC is one of the main evaluation indicators of the functional properties of dietary fiber, which could adsorb glucose molecules and delay the rise of blood glucose after meals, thus reducing the risk of diabetes (40). Results in Figure 7B suggested that the adsorption capacity of BIDF (10.73 mg/g) to glucose increased significantly (*P* < 0.05) after bagasse (6.47 mg/g) was treated with alkaline hydrogen peroxide. However, the glucose adsorption capacity of H-BIDF (5.58 mg/g) and E-BIDF (3.89 mg/g) decreased compared to BIDF (*P* < 0.05). This may be due to the destruction of the active site for glucose adsorption by multiple hydrothermal treatments.

#### 3.4.4. Bile salt adsorption capacity

The BSAC is commonly used as an index to assess the adsorption of lipophilic substances. On the one hand, dietary fiber with high BSAC can inhibit the absorption of cholesterol and promote its excretion (41). On the other hand, when the content of cholate decreases, the human body will automatically convert cholesterol into sodium cholate for supplementation, thereby promoting cholesterol consumption. A previous study (42) had shown that sugarcane fibers may bind sodium bile salts and our study subsequently confirmed it. Results in Figure 7C suggested that bagasse (2.43 mg/g) treated with AHP significantly increased the bile salt adsorption capacity of BIDF (8.24 mg/g) (*P* < 0.05), which indicated delignification of lignin was important to improve BSAC. H-BIDF (16.36 mg/g) and E-BIDF (15.86 mg/g) had a positive effect on improving BSAC, which may be due to their relatively high oil holding capacity or porous structure.

#### 3.4.5. Nitrite ion adsorption capacity

The NIAC is also a meaningful functional property of dietary fiber, which can usually prevent toxicity in humans following excessive nitrite intake (43). In this study, the NIAC of four fibers were measured at pH 2.0 and pH 7.0, respectively. The results showed that none of the four fibers had NIAC at pH 7.0, similar to that of wheat bran insoluble dietary fibers (44). As the picture depicted in Figure 7D, BIDF (222.74 μg/g) showed a stronger NIAC than bagasse (181.44 μg/g) at pH 2.0 (*P* < 0.05). Particularly, the H-BIDF demonstrated the highest NIAC (260.88 μg/g), which might be because of its wrinkled surface and the exposure of more functional groups. However, the NIAC of E-BIDF (182.02 μg/g) was decreased significantly (*P* < 0.05) after BIDF was hydrolyzed by hemicellulase, which may be due to the positive correlation between the size of NIAC and hemicellulose content.

## 4. Conclusions

In this study, response surface experiments were performed to obtain optimal AHP experimental conditions. The functional properties of BIDF were significantly enhanced compared to bagasse, indicating the importance of lignin removal for the modification of dietary fibers. Meanwhile, bagasse was modified using A-H and A-E treatments, respectively, and its modification effects were evaluated in terms of physicochemical, structural and functional properties to find a suitable modification method. The results showed that the structural integrity of the modified bagasse insoluble dietary fiber was disrupted and more folds or pores were formed, which could be responsible for the significant increase in its functional properties. Compared with BIDF, H-BIDF became less crystalline and part of the cellulose was degraded, which was also supported by FT-IR spectroscopy, resulting in a further increase of WHC, OHC, BSAC, and NIAC. Adsorption of water, oil, bile salts and nitrite ions can help fecal transport more comfortably and promote cholesterol degradation while taking away toxicogenic ions. It can be shown that A-H modification could be a guide to prevent constipation and obesity. On the contrary, the crystallinity and microscopic porosity increased after hemicellulase modification. Although WHC, OHC and NIAC of E-BIDF decreased, it obtained the highest CEC. High CEC creates a more durable environment for digestion and absorption by reversibly exchanging with organic cations, thus affecting the pH of the digestive tract. A-E modification was useful for the prevention of digestive tract diseases. In general, the modification methods were highly selective for improving physical, chemical and functional properties and can provide some guidance for the development of foods with specific functions. In the case of bagasse insoluble dietary fiber, A-H treatment was a better modification method.

## Data availability statement

The original contributions presented in the study are included in the article/supplementary material, further inquiries can be directed to the corresponding author.

## Author contributions

ML: data curation, writing—original draft, writing—review, editing, investigation, and formal analysis. ChengW: methodology, investigation, and formal analysis. ChensW: data curation, validation, formal analysis, and investigation. CX: supervision, project administration, and funding acquisition. FH: conceptualization, methodology, and resources. KL: conceptualization and resources. CS: visualization, investigation, and formal analysis. All authors have read and agreed to the published version of the manuscript.
